# Remote Sensing Inversion of Full-Profile Topography Data for Coastal Wetlands Using Synergistic Multi-Platform Sensors from Space, Air, and Ground

**DOI:** 10.3390/s25247405

**Published:** 2025-12-05

**Authors:** Jiabao Zhang, Jin Wang, Yu Dai, Yiyang Miao, Huan Li

**Affiliations:** 1 College of Harbour, Coastal and Offshore Engineering, Hohai University, Nanjing 210098, China; 2 Field Scientific Observation and Research Station of Ministry of Education for Tidal Flat Ecosystem in the Jiangsu Radial Sand Ridge Region, Hohai University, Nanjing 210098, China; 3 Suzhou Water Conservancy Project Administration, Suzhou 215004, China; 4 Nanjing Hezhen Lianxing Geographic Information Technology Co., Ltd., Nanjing 210017, China

**Keywords:** remote sensing, synergistic multi-sensor data, topographic inversion, Digital Elevation Model (DEM), tidal Flat, zonal fusion

## Abstract

This study proposes a “zonal inversion–fusion mosaicking” technical framework to address the challenge of acquiring continuous full-profile topography data in coastal wetland intertidal zones. The framework integrates and synergistically analyzes data from multi-platform sensors, including satellite, unmanned aerial vehicle (UAV), and ground-based instruments. Applied to the Min River Estuary wetland, this framework employs zone-specific optimization strategies: in the inundated zone, the topography was inverted using Landsat-9 OLI imagery and a Random Forest algorithm (R^2^ = 0.79, RMSE = 2.08 m); in the bare flat zone, a linear model was developed based on Sentinel-2 time-series imagery using the inundation frequency method, and it achieved an accuracy of R^2^ = 0.86 and RMSE = 0.34 m; and in the vegetated zone, high-precision topography was derived from UAV oblique photography with Kriging interpolation (RMSE = 0.10 m). The key innovation is the successful generation of a seamless full-profile digital elevation model (DEM) with an overall RMSE of 0.54 m through benchmark unification and precision-weighted fusion algorithms from the sensor data fusion perspective. This study demonstrates that the synergistic sensors framework effectively overcomes the limitations of single-sensor observations, providing a reliable and generalizable integrated solution for the full-profile topographic monitoring of tidal flats, which offers crucial support for coastal wetland research and management.

## 1. Introduction

Tidal flat wetlands are critical zones of land–sea interaction that provide essential ecosystem services, including biodiversity maintenance, carbon sequestration, and wave dissipation [1,2,3], through tidal-driven sediment transport and coupled vegetation–topography evolution. Due to increasing risks of tidal flat degradation [4,5,6], the development of high-spatiotemporal-resolution sensing monitoring technologies is imperative to support wetland conservation and sustainable management [7].

High-precision topography serves as the foundational element for tidal flat remote sensing monitoring, profoundly influencing tidal inundation processes, hydrodynamic distribution, and remote sensing signal transmission [8]. Traditional measurement methods are constrained by tidal windows and high costs, which poses challenges for large-scale dynamic monitoring [9]. Remote sensing technology has become the primary approach for topographic inversion due to its advantages of synoptic coverage and non-intrusive acquisition [10,11,12].

Recent international studies have advanced coastal wetland remote sensing through several key approaches: (1) refining inundation frequency and waterline methods for continuous topographic mapping in bare flats [13,14]; (2) fusing optical imagery with altimetry data from missions like SWOT and ICESat-2 to enhance retrieval accuracy [15]; (3) employing multi-dimensional time-series features for detailed wetland classification and geomorphic characterization [16,17].

The full-profile topography of tidal flats refers to the continuous topographic profile perpendicular to the shoreline from the seaward to the landward side [18,19]. Influenced by the combined effects of tidal–wave–runoff dynamics and vegetation coverage [5,20], tidal flat topography exhibits significant zonation. Thus, this study divides the tidal flat into three functional zones: inundated zone (permanently submerged areas), bare flat zone (exposed mud/sand flats with minimal vegetation), and vegetated zone (areas with high vegetation coverage like salt marshes) [21,22,23]. However, current studies remain largely confined to individual zones, resulting in fragmented inversion approaches [24] that hinder a comprehensive understanding of tidal flats as integrated geomorphological–ecological coupled systems. This limitation underscores the critical need for integrated frameworks that synergize multi-platform observations, as highlighted in recent studies [15].

Topographic inversion in the inundated zone is a key application of water depth remote sensing. Early methods were primarily based on physically driven models (e.g., linear, log-ratio) [25,26], but their application in optically complex waters is limited. In such environments, spectral coupling among the substrate, suspended sediments, and water constituents lead to ill-posed inversion problems [27]. In the past decade, a paradigm shift has occurred with the adoption of machine learning (e.g., Random Forest, neural networks), which implicitly captures complex non-linear mappings, thereby greatly improving accuracy in turbid waters [28,29].

Topographic inversion in the bare flat zone has evolved from discrete to continuous techniques. Traditional waterline methods construct DEMs by matching multi-temporal shoreline extractions with tidal data [30] but are limited by shoreline extraction accuracy and topographic discretization. Hydrodynamic model coupling corrects spatiotemporal differences in tidal wave propagation through numerical simulation [31] but involves a complex model setup and high computational cost. Airborne LiDAR achieves centimeter-level accuracy [32] but it is costly and less timely. The inundation frequency method, based on the “elevation–inundation frequency negative correlation” principle, uses long-term image series to statistically derive inundation frequency per pixel, establishing quantitative relationships with elevation for continuous mapping and showing great potential in large-scale estuarine and coastal studies [33,34].

The primary challenge in inverting topography in vegetated zones using optical remote sensing is signal distortion from canopy obstruction [35]. Current techniques include the following: airborne LiDAR extracting ground points via multiple returns [36], achieving decimeter-level accuracy but with high data acquisition costs; spaceborne laser altimetry systems penetrating partial vegetation [37], but are limited by vegetation density; while the penetration capability of synthetic aperture radar interferometry (InSAR) is highly dependent on the radar wavelength. While X-band InSAR exhibits limited penetration and is primarily suitable for sparse vegetation, longer wavelengths such as L-band and P-band (e.g., from the BIOMASS satellite) demonstrate significant penetration through vegetation canopies, showing greater potential for interferometric applications in densely vegetated areas [38,39,40]; meanwhile, deep learning-based algorithms showing promise for separating vegetation height and topography [41], but require large training samples and have limited generalization. The current trend involves developing multi-sensor synergistic inversion frameworks, integrating optical, laser, and radar sensing data advantages, combined with ground truth and UAV-based remote sensing surveys for multi-scale correction [42] to enhance accuracy and reliability for topographic inversion in the vegetated zone.

Despite these advancements for individual zones, research on cross-zone full-profile synergistic inversion remains scarce. This leads to the fragmentation of data information, hindering the interpretation of mechanisms for cross-zone material/energy exchange and overall evolution patterns [43]. The key challenges for full-profile inversion include the following: (1) The inherent differences in spatial resolution, sensor mechanisms, and accuracy among optimal data sources (e.g., satellite imagery, UAV sensing data) for different zones. (2) The error propagation and discontinuous boundaries during multi-source DEM mosaicking.

This study constructs and validates a “zonal inversion–fusion mosaicking” framework using the Min River Estuary as a case study. The key innovation lies in the systematic synergy of multi-platform sensors and zone-optimized inversion models, enabling the seamless integration of topographic information across heterogeneous zones. By developing benchmark unification and precision-weighted fusion algorithms, we overcome key mosaicking challenges to generate a continuous full-profile DEM. This framework provides a generalizable technical solution and essential baseline data for quantifying material fluxes and supporting system-scale coastal research and management.

## 2. Materials and Methods

**Figure 1:**
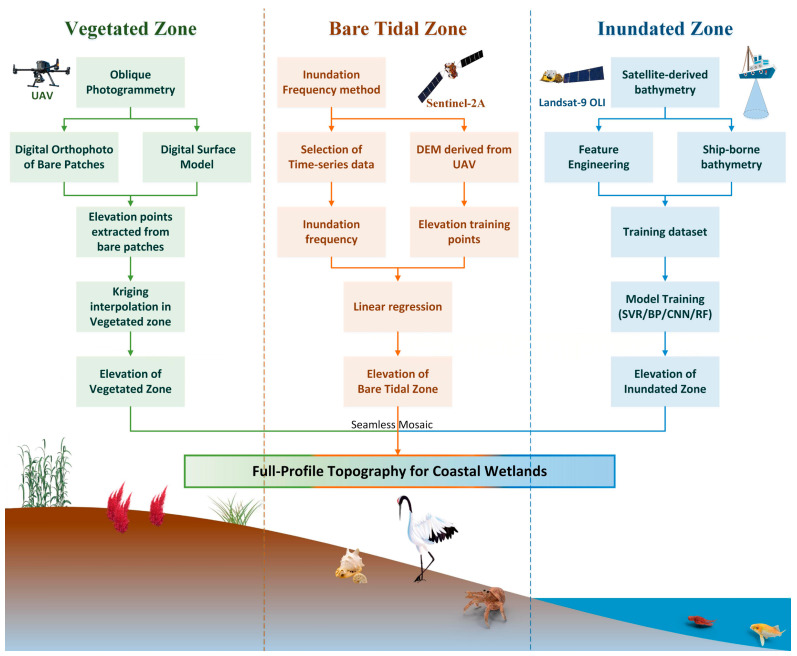
Study design flowchart for zonal inversion and fusion.

To address the challenge of acquiring continuous full-profile topography data in coastal wetlands, we designed a technical workflow (Figure 1) based on a “zonal inversion–fusion mosaicking” framework.

The workflow comprises three tailored components: (1) bathymetric inversion in the inundated zone using Landsat-9 OLI imagery and shipborne data through machine learning models (SVR, BP, CNN, RF); (2) elevation inversion in the bare tidal zone based on Sentinel-2A time-series imagery and the inundation frequency method; (3) terrain reconstruction in the vegetated zone via UAV oblique photogrammetry and Kriging interpolation of bare ground points. The three zonal DEMs are then integrated into a seamless full-profile product through benchmark unification and precision-weighted fusion algorithms.

### 2.1. Study Area

**Figure 2:**
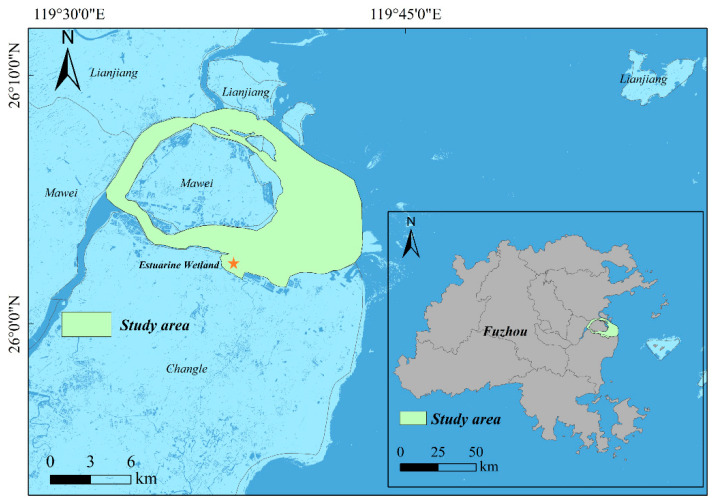
Location map of the study area. The star (★) denotes the location of the wetland park (estuary wetland) within the study area.

As shown in Figure 2, this study focuses on the intertidal zone of the Min River Estuary National Nature Reserve (geographical coordinates: 119°34′–119°40′ E, 26°02′–26°09′ N). The reserve covers approximately 2100 hectares, which includes a wetland park of about 281.85 hectares. The wetland park serves as an important habitat for migratory birds and aquatic organisms. It plays a crucial role in maintaining the ecological balance and environmental stability of the region. The mouth of the Min River is characterized by strong tidal currents, high suspended sediment concentrations, and intense river–sea interactions. These processes have shaped a typical shore-based zonation pattern consisting of perennial submerged channels, extensive tidal flats exposed between high tides, and salt marshes subjected to long-term inundation. The distinct functional zoning of “inundation–bare tidal area–vegetated area” corresponds to the concept of the “full-profile topographic inversion” method. This makes the estuary an ideal natural laboratory for developing and validating the proposed “zonal inversion–fusion mosaicking” framework.

### 2.2. Data Acquisition and Processing

#### 2.2.1. Spaceborne Sensors Data Acquisition and Processing

For topographic inversion in the inundated zone, Landsat-9 OLI multi-spectral imagery acquired on 15 September 2022 was employed, utilizing the Collection 2 Level-2 surface reflectance products. The Operational Land Imager (OLI) aboard Landsat-9 features nine spectral bands, with the visible to shortwave infrared bands (Bands 1–7) exhibiting a spatial resolution of 30 m, while the panchromatic band (Band 8) provides an enhanced spatial resolution of 15 m (NASA, 2021; USGS, 2024), and the cirrus detection band (Band 9) maintains 30 m resolution. These Level-2 products have been subjected to comprehensive atmospheric correction, where surface reflectance values are obtained through linear transformation of the pixel digital numbers [44].

The pre-processing involved three critical steps: (1) Projection transformation from the WGS84/UTM Zone 51N (WGS84: *World Geodetic System 1984*, and UTM: *Universal Transverse Mercator*) coordinate system to the WGS84 geographic coordinate system using ArcMap 10.7 (an ArcGIS tool) to unify the datums, thereby establishing the unification of spatial datums with shipborne bathymetric survey data. (2) Utilization of in situ bathymetric data that had been tidally corrected to the 1985 National Height Datum (as detailed below). (3) Land–water separation using the Normalized Difference Water Index (NDWI) for precise water body delineation, employing threshold-based segmentation to discriminate between aquatic and terrestrial pixels, which effectively eliminates land pixels to optimize the bathymetric inversion model’s specificity. The shipborne bathymetric data were corrected for tidal variations during the survey. The instantaneous water level was derived from Differential GPS (DGPS) ellipsoidal heights, transducer draft, and vessel motion parameters, and all depths were converted to the 1985 National Height Datum. This process established a consistent vertical reference. Therefore, the inversion model could learn a direct relationship between spectral features and datum-referenced depth. This made tidal correction of the satellite imagery unnecessary.

Topographic inversion in the bare flat zone was conducted using the European Space Agency’s Sentinel-2 Multi-Spectral Instrument (MSI) Level-2A surface reflectance products. The Sentinel-2 twin-satellite constellation offers a 5-day revisit cycle, with the MSI sensor encompassing 13 spectral bands. Among these, four visible to near-infrared bands (B2, B3, B4, B8) provide a spatial resolution of 10 m, six red-edge and shortwave infrared bands (B5, B6, B7, B8a, B11, B12) achieve 20 m resolution, while three atmospheric correction auxiliary bands (B1, B9, B10) maintain 60 m resolution.

The Level-2A products have undergone atmospheric and terrain correction through the Sen2Cor algorithm, providing bottom-of-atmosphere reflectance values [45]. To facilitate model development and synergistic validation with field-measured topographic data acquired on 20 September 2022, a Sentinel-2 scene with a proximate acquisition date (18 September 2022) was selected. For the bare flat zone, a time-series of 45 Sentinel-2 MSI Level-2A images (January 2020 to March 2024), with cloud cover under 15 percent, was utilized for inundation frequency analysis at a 10 m resolution. These images were radiometrically scaled to a 0–1 range and rigorously pre-processed using the quality assessment (QA) band to exclude pixels with a cloud probability exceeding 15%.

#### 2.2.2. UAV Oblique Photography Data and Processing

Topographic data for the vegetated zone were acquired using a DJI MATRICE 600 Pro hexacopter UAV platform on 20 September 2022. The platform was equipped with a Zenmuse X5R gimbal camera featuring a 4/3-inch CMOS sensor with an effective 16-megapixel resolution and a 15 mm fixed-focus lens, meeting the requirements for high-resolution coastal zone mapping. Flight parameters were configured as follows: flight altitude of 120 m, yielding a ground resolution of approximately 3 cm, with 80% forward overlap and 70% side overlap. To capture multi-angle geometric and textural information of surface features, a five-lens oblique photography configuration was employed, overcoming the limitation of traditional orthophotos that primarily capture top-down information. The flight mission was conducted during the ebb tide period to ensure full exposure of the tidal flat and avoid residual water effects on image quality; meteorological conditions included cloudy skies and wind speed < 5 m/s to minimize shadowing and distortion interference.

For the imagery collected by UAV oblique photography, three-dimensional reconstruction was performed using Structure from Motion (SfM) algorithms. The processing workflow comprised (1) image alignment, calculating camera exterior orientation elements through bundle adjustment and generating sparse point clouds; (2) dense matching, producing dense point clouds based on multi-view stereo vision algorithms; (3) mesh reconstruction, constructing a TIN model via Delaunay’s triangulation; (4) texture mapping, generating a Digital Surface Model (DSM) and Digital Orthophoto Map (DOM) with 0.05 m resolution.

**Figure 3:**
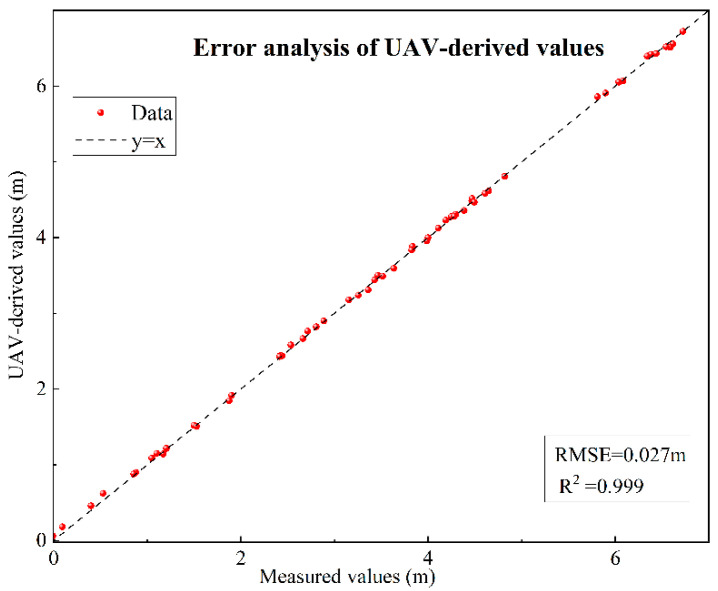
Error analysis of UAV-derived values.

Finally, comparative analysis between 56 RTK (Real-Time Kinematic)-measured elevation points and corresponding DSM-extracted values demonstrated a coefficient of determination (R^2^) of 0.999 and a Root Mean Square Error (RMSE) of 0.027 m, achieving centimeter-level accuracy. These high-precision validation results confirm that UAV-based oblique photography products can serve as reliable benchmark data for topographic inversion in vegetated zones, providing high-quality sample support for subsequent bare ground point extraction and Kriging interpolation. For the error analysis, linear regression was performed between the UAV-derived values and the measured values. The results demonstrate a very high agreement, as shown in Figure 3.

#### 2.2.3. Field Observation Transect Data

**Figure 4:**
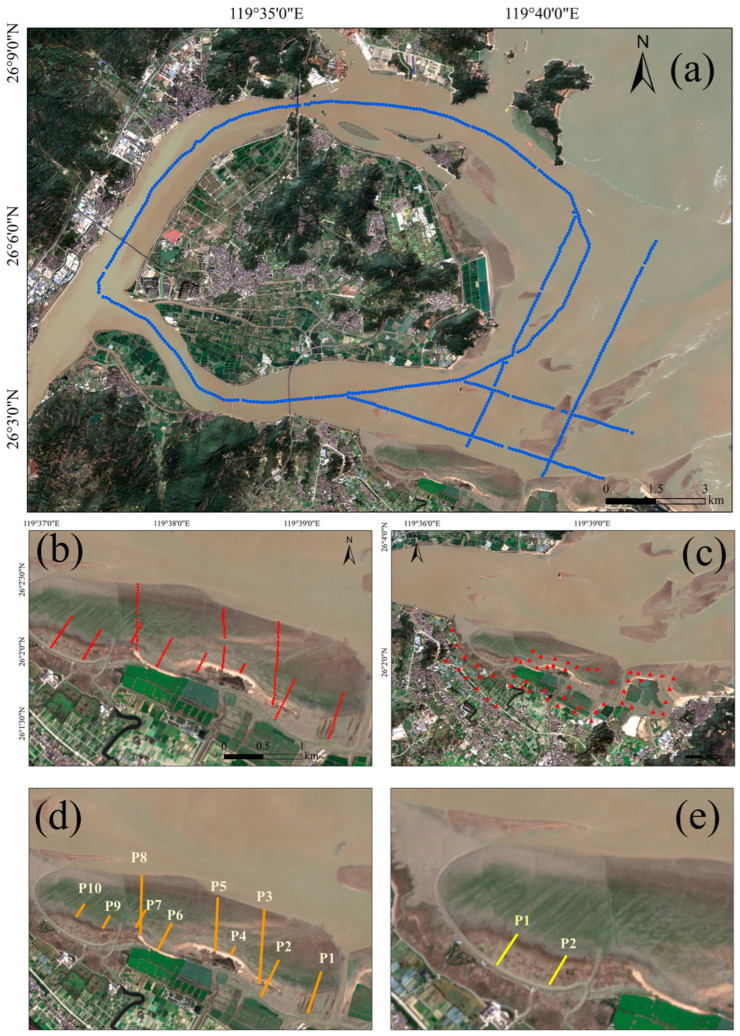
Field observation data. (**a**) Measured water depth points (blue dots). (**b**) Measured elevation values of tidal flats, including 161 measured elevation points (red dots) in the bare flat zone and 25 measured elevation points in the vegetation area. (**c**) UAV control points (solid red triangle). (**d**) Location of the 10 profiles used for validating the elevation inversion accuracy against measured values (for Figure 10). (**e**) Location of the 2 profiles used for comparing the interpolated results with measured values in the vegetated tidal flat (for Figure 12).

For the inundated zone, a shipborne echo sounder acquired 5 survey lines with 635 water depth points sampled at equal intervals, covering a depth range of 0–20 m, with data collected on 10 September 2022. For the bare flat and vegetated zones, GPS-RTK measurements obtained a total of 186 elevation points with an elevation range of −3 to 5 m, with data collected on 20 September 2022. The spatial distribution of all field observation points is shown in Figure 4.

### 2.3. Methods

#### 2.3.1. Topographic Inversion Method for the Inundated Zone

The essence of bathymetric inversion in the inundated zone is to establish a quantitative relationship between the apparent optical properties of the water body and the geometric water depth [46]. This study constructed a training sample set based on Landsat-9 OLI multi-spectral sensor data (Bands 1–7) and 629 shipborne bathymetry points (out of a total of 635 measured points, 6 were reserved for accuracy validation of the full-profile inversion results later in the text). Correlation analysis of band combinations indicated that four combined features B4 − B2 (r = 0.73), B3/B4 (r = −0.72), B4 + B5 (r = 0.71), and B3 × B5 (r = 0.71) exhibited significantly higher correlations with water depth than individual bands.

The high correlation coefficients (r > 0.71) of these band combinations with water depth served as the primary criterion for their inclusion, providing a preliminary basis for the feature engineering. The predictive utility of these selected features is subsequently assessed through a feature importance analysis.

Based on this, an 11-dimensional feature vector was constructed as the model input, comprising the original Bands 1–7 and the aforementioned 4 combined features, to maximize the retention of spectral information relevant to water depth. To address complex environments such as high suspended sediment and substrate heterogeneity in the estuary, this study constructed four non-linear machine learning models to explore the complex relationships between multi-dimensional spectral features and water depth, a strategy that is increasingly supported for complex aquatic environments [11].

Support Vector Regression (SVR): Utilized a radial basis function kernel to map the 11-dimensional input into a high-dimensional feature space. Kernel parameter γ = 0.8 and penalty factor C = 4.0 were optimized via grid search to balance model complexity and generalization ability.

BP Neural Network: Constructed a three-layer fully connected architecture (input layer: 11 nodes, hidden layer: 11 nodes, output layer: 1 node). Employed a momentum gradient descent algorithm (learning rate 0.01, momentum coefficient 0.09) to iteratively update weights through backpropagation, learning the complex non-linear mapping between input and output, incorporating an early stopping strategy to prevent overfitting.

Convolutional Neural Network (CNN): Designed a one-dimensional convolutional architecture to extract hierarchical representations of spectral features. It included two convolutional blocks (3 × 1 kernel, channels: 16→32), batch normalization layers, ReLU activation, and max pooling. Used an SGDM optimizer (initial learning rate 0.01, decaying to 0.001 after 800 epochs, maximum training epochs: 1200), leveraging local receptive fields and weight sharing mechanisms to extract spatial correlation patterns in spectral features.

Random Forest (RF): Based on an ensemble learning framework, constructed 91 decision trees, randomly selecting 5 features for each split. Utilized a Bagging mechanism and feature randomness to reduce variance. RF achieves a strong learner through the voting mechanism of multiple weak learners, offering inherent robustness to outliers, missing data, and small sample sizes, and requires no complex parameter tuning [47]. The optimal Random Forest model was applied to the Landsat-9 OLI imagery (15 September 2022) to generate a bathymetric Digital Elevation Model for the inundated zone at its native 30 m spatial resolution.

#### 2.3.2. Topographic Monitoring Method for the Bare Flat Zone

Topographic inversion in the bare flat zone was conducted using a time-series inundation frequency method. This study utilized Sentinel-2 Multi-Spectral Instrument (MSI) Level-2A surface reflectance products. A time-series of 45 cloud-filtered images (January 2020 to March 2024) was processed at the sensor’s native spatial resolution of 10 m. This method was selected for its optimal balance between spatial coverage, resolution, and cost, which aligns with the core objective of this study. While airborne LiDAR offers a superior accuracy, its expense hinders large-scale application [32,36]. The classical waterline method relies on discrete shorelines, lacking the pixel-level continuity of time-series approaches [30]. Satellite altimetry (e.g., ICESat-2, SWOT) provides direct measurements but suffers from sparse spatial sampling [10,37]. In contrast, the inundation frequency method cost-effectively leverages dense satellite archives to generate seamless, pixel-wise topographic models over extensive areas, which aligns with the core objective of this study. The applicability and refinement of this approach for large-scale tidal flat mapping have been further demonstrated in recent studies [13,42].

The inundation frequency method was selected for its optimal balance between accuracy and feasibility, a choice well supported by the Min River Estuary’s hydro-geomorphic setting [16]. The large tidal range and regular semi-diurnal tides provide a stable hydrodynamic basis, while the gentle slope of the bare flat satisfies the method’s key assumption of a monotonic elevation–frequency relationship. This time-series approach effectively mitigates stochastic noise (e.g., from wind waves) inherent in single-scene waterline methods and cost-effectively generates seamless, pixel-wise topographic models over large areas, aligning with our core objective.

The method operationalizes the principle of a “negative correlation between elevation and inundation frequency” [48]. By statistically converting long-term submergence data from the temporal domain into spatial elevation information [46,49], it achieves continuous topographic mapping of the bare flat. The local tidal dynamics create ideal conditions for a stable frequency–elevation relationship, though accuracy can diminish where tidal flow is obstructed [13].

To enhance the distinction between water and bare ground in the tidal flat area, the Modified Normalized Difference Water Index (MNDWI) [50] was used, which is calculated as follows (Formula (1)):

(1) $$MNDWI=\frac{\rho_{Green}-\rho_{SWIR}}{\rho_{Green}+\rho_{SWIR}}$$

where Green represents reflectance in the green band, corresponding to Band 3 (B3) in Sentinel-2, and SWIR represents reflectance in the shortwave infrared band, corresponding to Band 11 (B11) in Sentinel-2. Typically, a pixel is highly likely to be water if MNDWI > 0; conversely, if MNDWI < 0, the pixel is more likely to be bare ground or vegetation. Compared with the traditional NDWI, MNDWI effectively suppresses interference from vegetation and salt marsh in water extraction, making it particularly suitable for water identification and inundation frequency calculation in tidal flat wetlands.

Based on the GEE platform, a collection of Sentinel-2 images from January 2020 to March 2024 with cloud cover < 15% was loaded. Reflectance was first scaled to the 0–1 range. Then, MNDWI was calculated for each scene, and water–land binary classification was performed using a threshold of 0.05. Pixels with MNDWI > 0.05 were classified as inundated and assigned a value of 1; otherwise, they were assigned 0. This threshold of 0.05 was empirically determined to optimally separate water from bare sediment in the tidal flat environment. The inundation status across all images in the time-series was statistically summed for each pixel, yielding the total number of inundations and the total number of valid observations. The ratio of these two values is the inundation frequency (F), which for each pixel is then calculated as follows (Formula (2)):

(2) $$F_{inundation}=\frac{N_{inundation}}{N_{observation}}$$

where N_inundation is the total number of inundated observations, and N_observation is the total number of valid observations. F ranges from 0 to 1, where 0 indicates never inundated (highest elevation) and 1 indicates always inundated (lowest elevation).

Based on 2960 high-precision bare flat elevation points acquired from UAV photogrammetry, the corresponding inundation frequency values were extracted to construct an inundation frequency–elevation sample dataset. A linear regression model was then calibrated. The model’s performance was rigorously evaluated against the UAV benchmark data using the coefficient of determination (R^2^), Root Mean Square Error (RMSE), and Mean Absolute Error (MAE). The gentle slope and low micro-topographic relief of the bare flat ensure a strong monotonic relationship between inundation frequency and elevation, providing a solid foundation for the linear model. A linear regression model was established to reveal the quantitative relationship between the two variables. The 10 m inundation frequency map was calibrated against a high-precision UAV-derived DEM (2960 points) to establish a linear inversion model, producing a continuous 10 m topographic model for the bare flat zone.

#### 2.3.3. Topographic Monitoring Method for the Vegetated Zone

As the Digital Surface Model (DSM) obtained from UAV oblique photography in the vegetated zone records the elevation at the top of the vegetation canopy rather than the true ground elevation, this study employed a bare point spatial interpolation method for DEM reconstruction in the vegetated area. This method involves extracting deterministic ground points from the DSM and using spatial interpolation algorithms to restore the continuous terrain surface, offering advantages of controllability and suitability for complex vegetation cover environments [51].

Bare ground point extraction followed a quantitative, two-step protocol applied to the UAV-derived DSM and DOM to ensure objectivity and reproducibility: (1) Spectral–Textural Screening: Candidate points were identified by targeting pixels on the DOM exhibiting the homogeneous, low-reflectance texture characteristic of exposed sediment, while explicitly excluding areas with vegetation canopy, water bodies, or floating debris. (2) Quantitative Elevation Filtering: The elevation of each candidate point was validated against the local median elevation calculated from its immediate 3 × 3 pixel neighborhood in the DSM. Any point exhibiting an absolute elevation deviation greater than 0.15 m was rejected as an outlier (e.g., misclassified micro-features like tidal creek scarps or vegetation roots) [52]. This threshold-based filtering ensured the objectivity and reproducibility of the sample set.

Based on the sample points, spatial interpolation was performed using the Ordinary Kriging geostatistical method. Kriging interpolation, based on the theory of regionalized variables, provides optimal unbiased estimation for unknown locations by analyzing the spatial autocorrelation structure of the sample points. Compared with deterministic interpolation methods like Inverse Distance Weighting, it is more suitable for capturing the spatial continuity and local variation characteristics of terrain. This method models spatial dependence through a semi-variogram, adaptively determining weight coefficients based on the spatial distribution characteristics of the sample points, achieving optimal prediction of elevation at unknown points. Interpolation parameters were set as follows: variable search radius strategy, 12–15 neighborhood samples, spherical model for the semi-variogram, and an output resolution of 0.5 m. A Digital Terrain Model for the vegetated zone was generated from UAV oblique photography (20 September 2022). A total of 23,732 bare ground points were extracted and interpolated using Ordinary Kriging, yielding a high-resolution DEM of 0.5 m, validated with an RMSE of 0.10 m against RTK measurements. The interpolation results generated a continuous DEM for the vegetated zone, clearly depicting terrain undulations, tidal creek pathways, and slope break lines, effectively restoring the true landform beneath the vegetation cover.

## 3. Results

### 3.1. Topographic Inversion Results and Accuracy Assessment for the Inundated Zone

To evaluate the performance of different machine learning algorithms for bathymetric inversion in an optically complex estuarine environment, this study constructed four multi-factor inversion models: SVR, BP, CNN, and RF. Based on shipborne measured water depth sample points, the four models were systematically trained and tested. The accuracy evaluation results are shown in Table 1.

**Figure 5:**
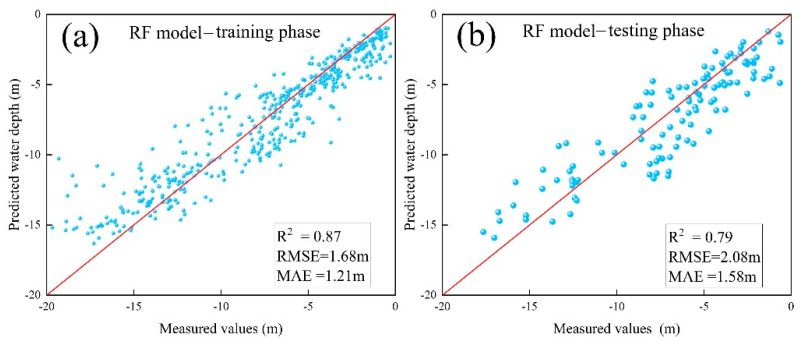
Accuracy of RF neural network model. (**a**) Training accuracy. (**b**) Testing accuracy.

The accuracy assessment showed that all four models effectively established non-linear mapping relationships between multi-spectral features and water depth, but their performance varied. The Random Forest model performed the best, achieving an inversion accuracy of R^2^ = 0.79, RMSE = 2.08 m, and MAE = 1.58 m within the 0–20 m water depth range (as shown in Figure 5). RF demonstrated a superior performance, achieving a 12.8% higher R^2^ and an 18.2% lower RMSE compared with other models. This advantage primarily stems from the ensemble learning mechanism of Random Forest, which effectively handles the spectral complexity caused by high suspended sediment concentrations and substrate heterogeneity in the estuary. The voting mechanism of the 91 decision trees reduces the risk of model overfitting, and feature randomness enhances robustness to noisy data.

Figure 5 presents the relationship between the predicted water depths and measured water depths for the RF model during the training and testing phases. The predicted water depths are well aligned with the measured values (R^2^ = 0.87 and 0.79 for training and testing, respectively).

**Figure 6:**
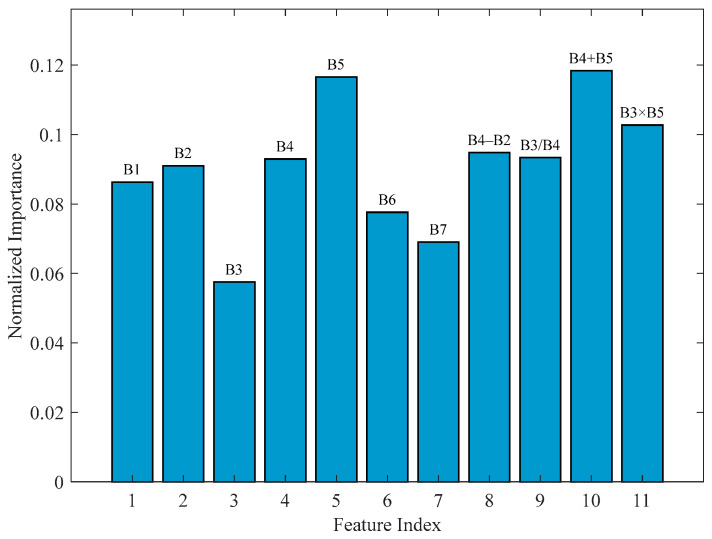
Feature importance ranking of the RF bathymetry model.

The feature importance analysis of the optimal RF model provided a critical evaluation of the feature engineering strategy. As illustrated in Figure 6, the results demonstrated that the composite feature B4 + B5 and the red band B5 were among the most influential predictors, which aligns with their known sensitivity to water optical properties. This agreement corroborates the initial feature selection based on correlation analysis.

Model accuracy exhibited a depth-dependent trend, with an excellent performance in shallow waters but a slight systematic underestimation observed for depths up to ~15 m. This aligns with the known challenge of optical signal attenuation in deep, turbid waters [27]. This fundamental limitation of optical bathymetry [24,53] could be mitigated in future work by integrating active sensors such as ICESat-2 [46]. Nevertheless, the model effectively reconstructed the overall topographic profile, including the main navigation channel, thus fulfilling the primary inversion objective. Our Random Forest model’s performance is comparable to the CNN-based Tide2Topo [54], validating its efficacy. This contrasts with paradigms prioritizing tidal stage selection, such as LogiTide2DEM [14].

**Figure 7:**
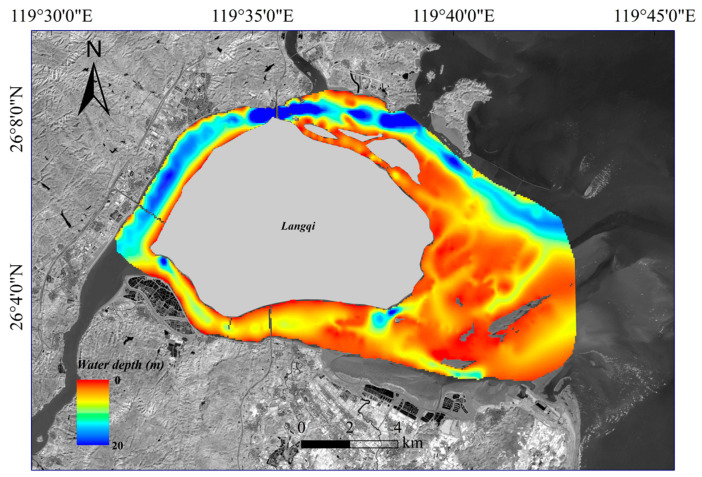
Bathymetric map of the inundated zone derived from the RF model.

Based on the optimal RF model, bathymetric inversion was performed for the inundated zone of the Min River Estuary (Figure 7). The inversion results clearly reveal the estuarine geomorphological pattern of alternating deep channels and shallow areas. The main navigation channel reaches depths of 15–20 m, exhibiting significant tidal dynamic erosion features. The water depth in the shallow areas on both sides is mostly 3–8 m, with a gentle topographic transition. The water depth in the estuary mouth area shows a decreasing trend along the course, influenced by the interaction of runoff and tidal currents.

### 3.2. Topographic Inversion Results and Accuracy Assessment for the Bare Flat Zone

**Figure 8:**
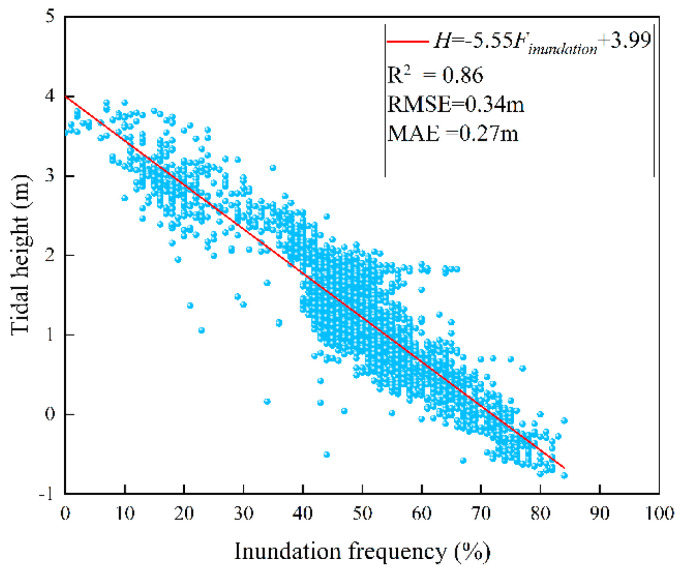
Linear regression model between inundation frequency and tidal flat elevation.

For the topographic inversion in the bare flat zone, this study calculated areal inundation frequency raster data based on the GEE platform. Combined with 2960 high-precision ground control points obtained from UAV oblique photography, their corresponding inundation frequency values were extracted to construct an elevation-paired dataset. After comparing and analyzing the relationship between inundation frequency and measured elevation, a linear inversion model Elevation = a × F + b was established. The model accuracy is shown below. A significant negative correlation was observed between inundation frequency and bare flat elevation (R2 = 0.86, RMSE = 0.34 m, MAE = 0.27 m; Figure 8), demonstrating the model’s capability to accurately characterize topographic variations.

**Figure 9:**
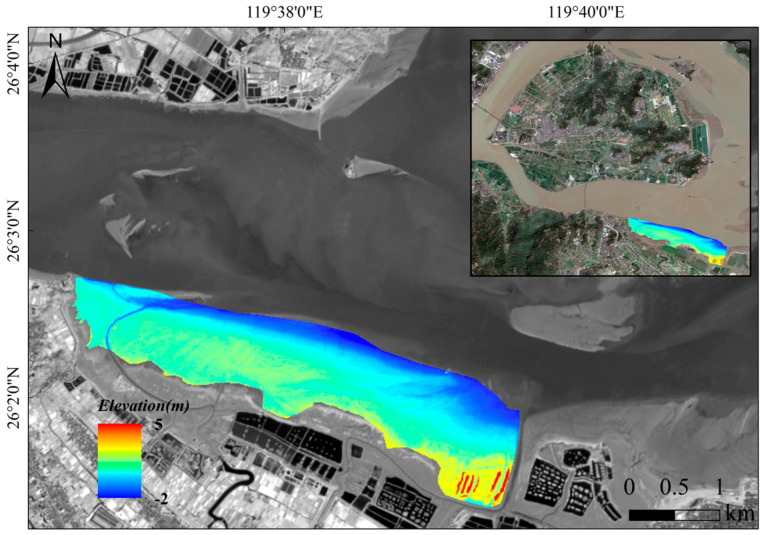
Topographic map of the bare tidal flat derived from the linear inversion model.

Based on this linear relationship, a continuous topographic elevation map (Figure 9) of the bare flat zone in the Min River Estuary was generated.

**Figure 10:**
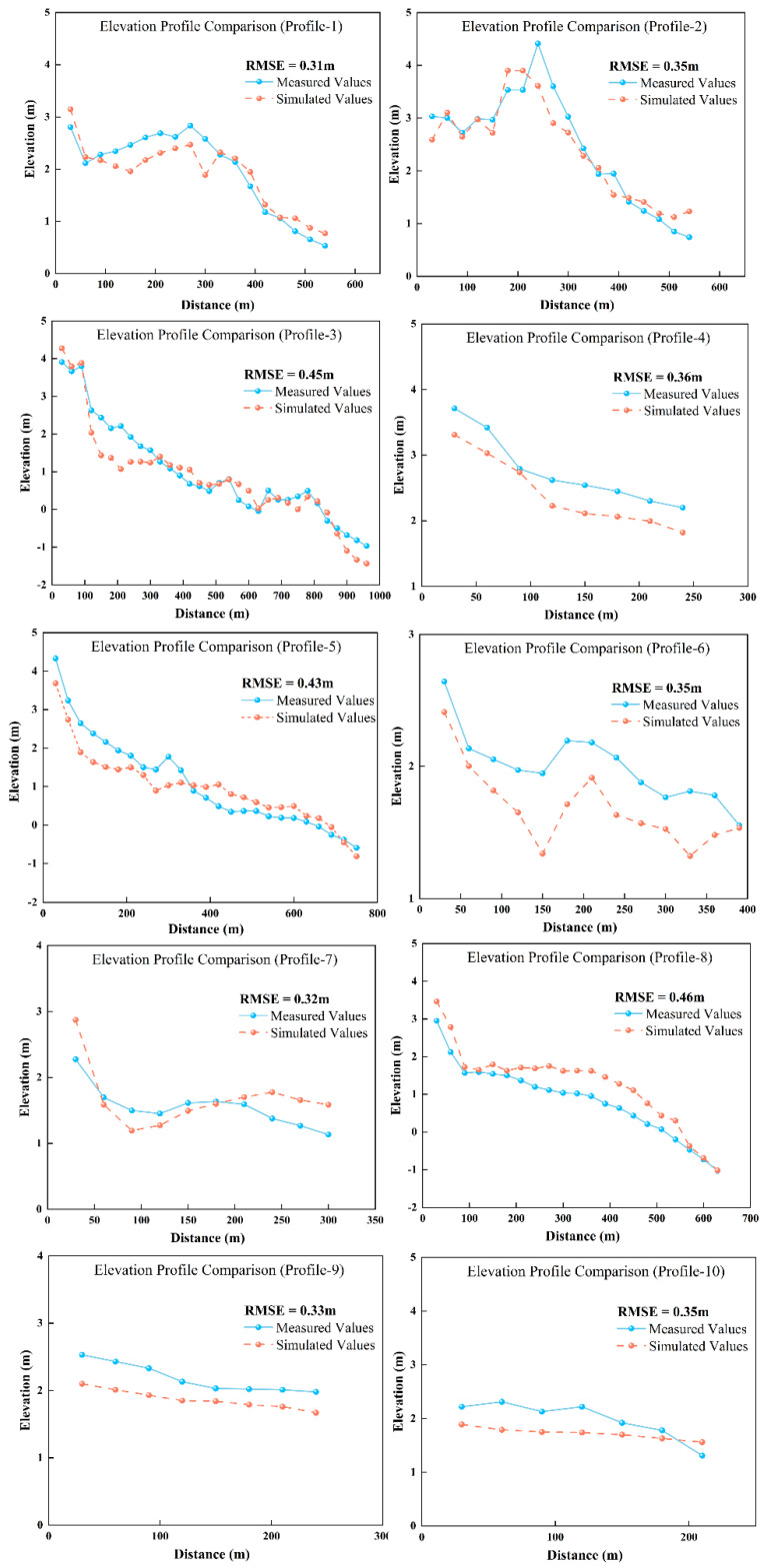
Comparison of measured and inverted elevation values. The locations of the profiles are shown in Figure 4.

To validate the accuracy of the inversion results, this study used 10 vertical transects measured in October 2022 for independent validation. The results show good overall agreement between the inverted elevations and the measured elevations, with consistent morphological trends across all transects (Figure 10). This indicates the high reliability of the linear inversion method based on inundation frequency for estimating bare flat topography. Errors varied somewhat between different transects: the maximum Root Mean Square Error occurred in transect 8 (0.46 m), and the minimum in transect 1 (0.31 m). The average Root Mean Square Error across the 10 transects was 0.37 m, indicating small overall inversion errors that meet the accuracy requirements for analyzing tidal flat topographic changes.

### 3.3. Topographic Inversion Results and Accuracy Assessment for the Vegetated Zone

Topographic inversion in the vegetated zone employed a method combining bare point extraction and Kriging interpolation. High-confidence bare ground sample points were identified through visual interpretation from the high-resolution DSM and DOM generated by UAV oblique photography. Based on a comprehensive consideration of spectral–textural features and elevation rationality, a total of 23,732 high-quality bare ground sample points were extracted, with a uniform spatial distribution covering geomorphic units such as tidal creeks, slopes, and platforms. Spatial interpolation was performed across the entire area using the Ordinary Kriging geostatistical method, generating a continuous DEM for the vegetated zone.

**Figure 11:**
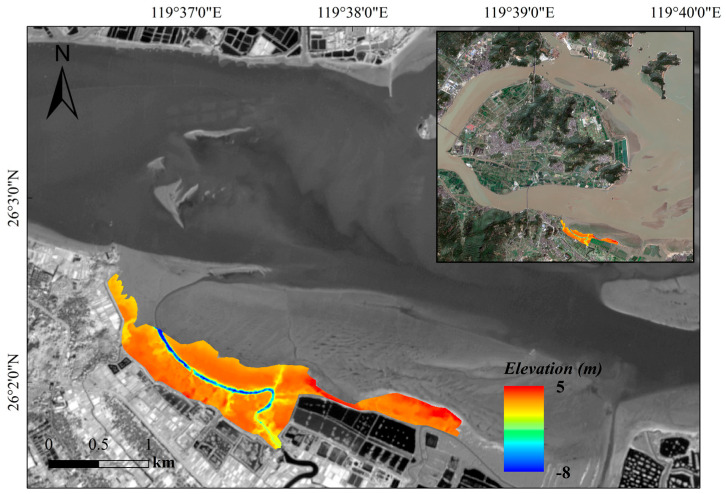
Topographic map of the vegetated zone derived from interpolation of UAV oblique photography.

The inversion results clearly present the complex topographic features of the vegetated zone. The tidal creek system is fully developed with clearly discernible pathways, with elevation ranges between −5 m and −8 m, forming the main drainage network of the vegetated area. Using the main tidal creek as the geomorphic boundary, the terrain in the southern and western areas is relatively high, with elevations mainly distributed between 2.5 m and 3.8 m, showing a trend of gradual uplift from the tidal creek inland, where elevation increases with distance from the creek. The area north of the tidal creek has elevations between 1.8 m and 3.2 m, showing a spatial pattern of gradual decrease towards the sea. The elevation significantly increases at the western edge of the vegetated zone, mainly distributed between 3.5 m and 4.9 m. The overall terrain has gentle relief but distinct micro-topographic differentiation, with slope break lines clearly outlining the boundaries of different geomorphic units, indicating that Kriging interpolation effectively restored the true land surface under vegetation cover (Figure 11).

**Figure 12:**
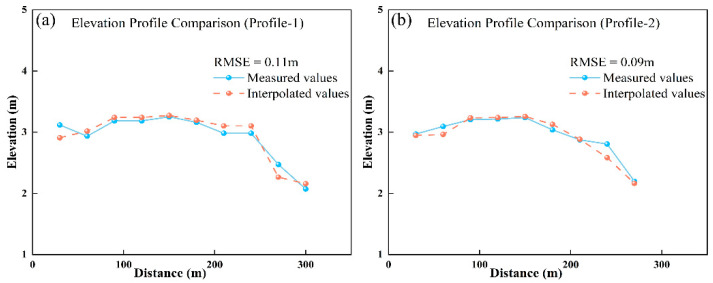
Comparison of interpolated and measured values in the vegetated tidal flat.

To verify the reliability of the interpolation results, an accuracy assessment was conducted using 19 measured terrain points (distributed along two vertical transects) collected in September 2022. By extracting the interpolated elevation values at the corresponding locations of the measured transects and comparing them with the measured values, the statistical analysis showed RMSE values of 0.11 m and 0.09 m for the two transects, with an average RMSE of 0.10 m (Figure 12).

### 3.4. Full-Profile Topographic Inversion Results

**Figure 13:**
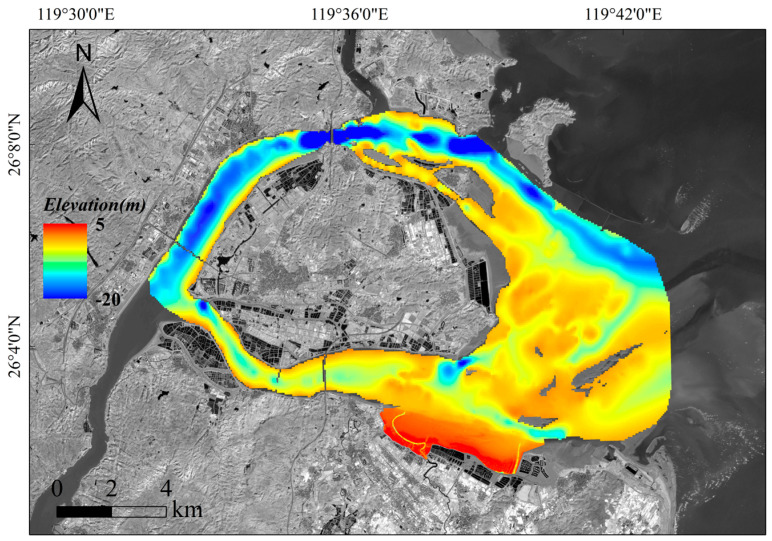
Min River Estuary full-profile DEM mosaic (from multi-platform data).

A seamless full-profile Digital Elevation Model (DEM) of the Min River Estuary, integrating the vegetated zone, bare flat, and inundated zone, was successfully generated (Figure 13), demonstrating the effective implementation of the “zonal inversion–fusion mosaicking” technical framework. The fusion procedure was systematically conducted as follows:

(1) Data Pre-processing and Datum Unification: All zonal DEMs were transformed to a unified WGS84/UTM projection coordinate system and resampled. Elevation datum corrections, calibrated against RTK control points, were applied to eliminate systematic biases. (2) Precision-Weighted Fusion Algorithm: A weighted feathering mosaicking method was employed. In overlapping areas, a precision-weighted fusion algorithm was applied, assigning greater weights to the high-accuracy data from the bare flat zone (RMSE = 0.34 m) and vegetated zone (RMSE = 0.10 m). (3) Seamless Transition Processing: An appropriate feathering width was configured for blending mode implementation, ensuring smooth transitions between different data sources within the transition zone and maintaining geomorphological continuity. (4) Quality Control and Iterative Optimization: Slope analysis and profile extraction methods were used to check for anomalous steep slopes or topographic discontinuities in boundary areas. Iterative adjustments to feathering parameters were performed until the geomorphological continuity requirements were satisfied.

**Figure 14:**
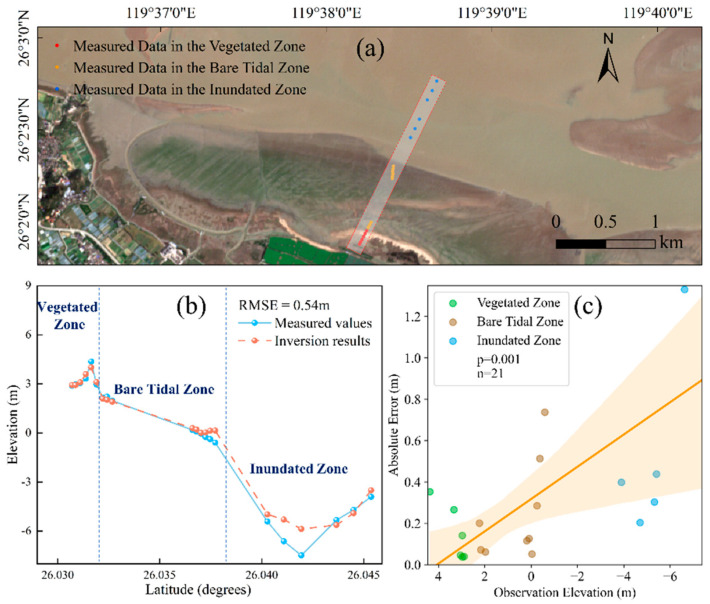
Accuracy validation of the integrated full-profile DEM. (**a**) Distribution of field validation points across the three functional zones. (**b**) Comparison between measured and inverted elevation values. (**c**) Scatter plot of observation elevation versus absolute error.

To quantitatively validate the full-profile mosaicking accuracy, a representative transect spanning the three functional zones was selected. The corresponding inverted elevation values for 21 measured points (6 in the vegetated zone, 9 in the bare flat zone, and 6 in the inundated zone) were extracted for comparative analysis. The statistical results show that the full-profile DEM achieved an overall RMSE of 0.54 m (Figure 14b). A zone-specific breakdown of the validation accuracy further elucidates the fusion performance: the RMSE for points within the vegetated zone was 0.10 m, the bare flat zone was 0.34 m, and the inundated zone was 2.08 m. The fact that the overall accuracy (0.54 m) effectively intermediates between the high-precision zones and the lower-precision inundated zone robustly demonstrates that the precision-weighted fusion algorithm successfully contained error propagation. A point-by-point analysis reveals a systematic increase in estimation uncertainty with water depth (Figure 14c), with errors below 0.35 m in the emergent zones but rising substantially in the subtidal area.

This analysis of the error distribution confirms a fundamental trend: estimation uncertainty increases systematically with water depth (Figure 14c), underscoring the inherent challenge of remote sensing bathymetry inversion in an optically complex inundated zone. This integrated DEM provides a reliable foundation for coastal geomorphological research and management.

## 4. Discussion

### 4.1. Applicability and Limitation of the Inundation Frequency Method for the Bare Flat Zone

The linear inversion model based on the negative correlation between inundation frequency and elevation achieved good inversion results in the mid- to low-tidal flat areas. For the few steep areas in the eastern part of the bare flat zone, supplementation with UAV survey data was used for optimization. The applicability of the inundation frequency method is closely related to the range of tidal dynamic control. This method performs excellently in areas with significant periodic tidal action. However, when the elevation exceeds the local tidal control range in the Min River Estuary, the sparsity of inundation events affects the inversion accuracy to some extent. Uncertainty also increases at tidal creek edges or where structures disrupt flooding [13], suggesting a need to incorporate geomorphological metrics [17]. Potential future directions include the exploration of segmented modeling strategies that incorporate tidal-level probability distributions, as well as the integration of multi-source satellite data to increase temporal coverage density—both of which would expand the method’s applicability and enhance inversion accuracy. This comparative perspective ultimately positions the inundation frequency method as a powerful tool for regional-scale assessment and dynamic monitoring, particularly in data-scarce environments. For applications demanding centimeter-level accuracy or targeting micro-topographic features, techniques like LiDAR or UAV surveys remain indispensable [32,51]. The choice of method should therefore be guided by the specific spatial scale and precision requirements of the intended application.

### 4.2. Key Issues in Vegetated Zone Topographic Reconstruction

For topographic reconstruction in the vegetated zone, the accuracy and density of high-confidence bare ground point extraction are limiting factors. Remote sensing signals in vegetated areas are complex; bare points extracted based on spectral features are susceptible to influences from vegetation type, coverage, and phenology, posing a risk of misclassification. This study comprehensively utilized UAV survey data and adopted the Kriging interpolation method for topographic reconstruction. The interpolation accuracy was good in vegetated areas with a sufficient distribution of bare points, but it may decrease in densely vegetated areas due to the relative sparseness of bare points. Future work should increase the density of measured topographic points in the vegetated zone, utilize the canopy-penetrating capability of LiDAR or longer-wavelength SAR data (e.g., L-band) to obtain ground elevation, or develop deep learning-based inversion algorithms for vegetated areas to improve the accuracy of topographic reconstruction in vegetation-covered regions.

### 4.3. Challenges and Solutions for Multi-Source DEM Seamless Mosaicking

The seamless mosaicking of multi-source DEMs is a key link in obtaining full-profile topography data for coastal wetlands. This process represents a common and significant challenge in multi-sensor data integration, as discussed in recent reviews [42]. Differences in spatial resolution and grid alignment between the inundated zone’s Landsat-9, the bare flat zone’s Sentinel-2, and the vegetated zone’s UAV oblique photography data lead to geometric misalignment. Inconsistent elevation datums among different data sources can create step effects at boundaries; direct mosaicking may produce false topographic breaks, damaging the natural integrity of the topography. Our “zonal inversion–fusion mosaicking” framework is designed to systematically address these challenges, moving beyond the limitations of single-zone studies [17] by integrating multi-platform data, even as emerging techniques utilizing new sensors like SWOT [15] or ICESat-2/Sentinel-2 fusion [10,52] continue to evolve. To address these issues, this study projected the three zones to the WGS84/UTM system and resampled them at the level of datum unification, while establishing elevation datum transformation parameters through least squares adjustment to eliminate systematic bias. In the design of the mosaicking algorithm, an adaptive strategy based on geomorphic units was adopted, constructing transition zones extending from the central boundary line towards both sides, applying a precision-weighted fusion algorithm to give a greater weight to high-precision data, combined with feathering processing to eliminate step effects. Nevertheless, these methods still face challenges such as the lack of uniform standards for setting the mosaicking belt width and slight topographic deformation caused by uneven sensor-derived errors across regions. Subsequent research could explore directions, including integrating multi-dimensional features to automatically extract the optimal mosaicking path, constructing a spatiotemporal dynamic mosaicking framework coupled with tidal models to generate corresponding DEMs, in order to reduce mosaicking difficulty and errors, and enhance the model’s transferability and application value.

The validation of the seamless DEM, while confirming overall consistency, is constrained by the limited number of sample points (*n* = 21) along a single transect. This density is inadequate for resolving micro-topographic variations, especially at critical zone interfaces. Conducting a dense validation survey across these transitions is a crucial next step.

## 5. Conclusions

To overcome the challenges of multi-platform sensor data heterogeneity and methodological fragmentation in generating full-profile intertidal topography data, this study developed a “zonal inversion–fusion mosaicking” framework, using the Min River Estuary National Nature Reserve as a case study. The technical framework successfully achieved the high-precision inversion of a continuous topographic profile across the intertidal flat. Its innovation lies in the systematic integration and intelligent fusion of space–air–ground multi-sensor data, which leverages the respective advantages of the different platforms across various zones.

In the inundated zone, based on Landsat-9 OLI imagery, an 11-dimensional feature vector comprising Bands 1–7 and their combinations was constructed. Four machine learning models—SVR, BP neural network, CNN, and Random Forest—were systematically compared. The optimal Random Forest algorithm was selected to construct the bathymetric inversion model, achieving an inversion accuracy of R^2^ = 0.79 and RMSE = 2.08 m within the 0–20 m water depth range, effectively handling the spectral complexity in the high suspended sediment estuarine environment.

In the bare flat zone, utilizing 45 Sentinel-2 images from 2020 to 2024 acquired via the GEE platform, the MNDWI was calculated from time-series and converted into inundation frequency. A linear inversion model between inundation frequency and elevation was established, achieving an inversion accuracy of R^2^ = 0.86 and RMSE = 0.34 m within the −2~4 m range, thereby realizing full-coverage continuous mapping of the mid-to-low tidal flat area.

In the vegetated zone, high-resolution DSM and DOM were acquired using UAV oblique photogrammetry. Through visual interpretation, 23,732 high-confidence bare ground sample points were extracted. Topographic elevation was reconstructed using Kriging interpolation, achieving a decimeter-level accuracy with RMSE = 0.10 m in areas with a sufficient bare point distribution.

Following the zonal inversions, seamless DEM mosaicking was accomplished by unifying the projection and raster resolution, designing adaptive mosaicking zones based on geomorphic units, and applying precision-weighted fusion algorithms, ultimately obtaining the full-profile topography of the Min River Estuary coastal wetland.

The technical framework established in this study overcomes the limitations of traditional single-zone inversion, creating a paradigm for multi-sensor collaboration and intelligent fusion. It provides reliable, full-profile topographic baseline data for analyzing hydrodynamic processes, assessing biological habitats, and supporting coastal zone management in tidal flat systems. This study provides a valuable methodological reference and has significant practical implications for ecological environment monitoring, oceanographic research, and the sustainable development of analogous estuarine and coastal zones worldwide.

## Figures and Tables

**Table 1:** Evaluation of the accuracy of the bathymetry inversion model.

Model Type	R^2^	RMSE (m)	MAE(m)
SVR	0.62	2.91	2.20
BP	0.73	2.47	1.74
CNN	0.75	2.25	1.77
RF	0.79	2.08	1.58

## Data Availability

The data presented in this study are available on request from the corresponding author (The satellite imagery data used in this study is publicly available. The UAV, RTK, and bathymetric data are owned by the authors’ institution, and any sharing is contingent upon formal approval from the institution).
